# A Review of Ku-Band GaN HEMT Power Amplifiers Development

**DOI:** 10.3390/mi15111381

**Published:** 2024-11-15

**Authors:** Jihoon Kim

**Affiliations:** School of Electronic Engineering, Kyonggi University, Suwon-Si 16227, Republic of Korea; j7h7@kgu.ac.kr; Tel.: +82-31-249-9803

**Keywords:** Ku-band, GaN HEMT, high-power amplifier, satellite communications, MMIC, IM-PAM

## Abstract

This review article investigates the current status and advances in Ku-band gallium nitride (GaN) high-electron mobility transistor (HEMT) high-power amplifiers (HPAs), which are critical for satellite communications, unmanned aerial vehicle (UAV) systems, and military radar applications. The demand for high-frequency, high-power amplifiers is growing, driven by the global expansion of high-speed data communication and enhanced national security requirements. First, we compare the main GaN HEMT process technologies employed in Ku-band HPA development, categorizing the HPAs into monolithic microwave integrated circuits (MMICs) and internally matched power amplifier modules (IM-PAMs) and examining their respective characteristics. Then, by reviewing the literature, we explore design topologies, major issues like oscillation prevention and bias circuits, and heat sink technologies for thermal management. Our findings indicate that silicon carbide (SiC) substrates with gate lengths of 0.25 μm and 0.15 μm are predominantly used, with ongoing developments enabling MMICs and IM-PAMs to achieve up to 100 W output power and 30% power-added efficiency. Notably, the performance of MMIC power amplifiers is advancing more rapidly than that of IM-PAMs, highlighting MMICs as a promising direction for achieving higher efficiency and integration in future Ku-band applications. This paper can provide insights into the overall key technologies for Ku-band GaN HPA design and future development directions.

## 1. Introduction

**Figure 1:**
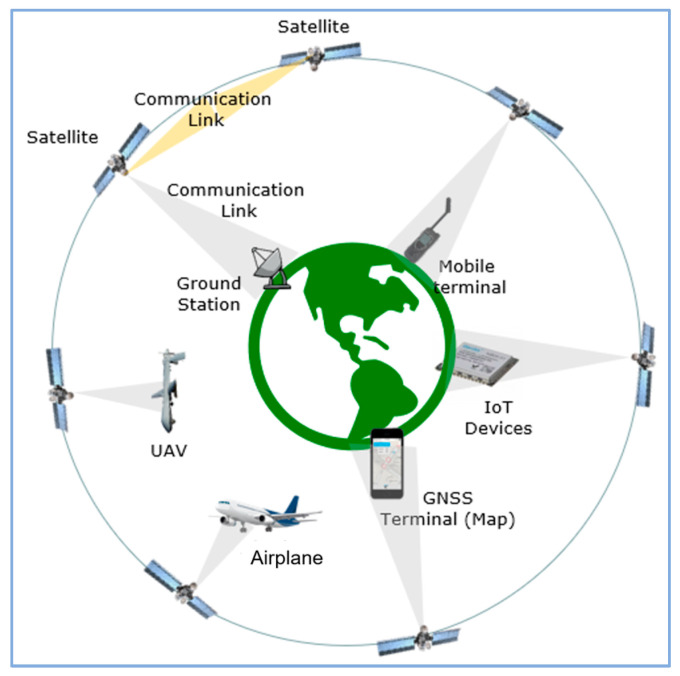
Various applications of satellite communications. Figure reproduced from [4].

In recent years, the demand for satellite communication services has increased not only in military applications but also in civilian ones, drawing significant attention to related technology development. Satellite communication technology enables communication between earth stations via satellites, providing various communication and broadcasting services [1,2,3]. Figure 1 shows the various applications of satellite communications [4]. Satellite communication can be classified into low, medium, and high orbits, including geostationary orbits, based on altitude. Geostationary satellite communication can cover the entire globe with just three satellites, offering the economic advantage of replacing numerous ground base stations for wide coverage. In contrast, low-orbit satellite communication provides high-speed communication with low latency. In particular, the recent Russian–Ukrainian war and the frequent occurrence of various disasters have highlighted the advantages of maintaining communication during emergencies and crises, leading to the emergence of private satellite communication companies such as Starlink. In addition to civilian communication services, there is a growing demand for satellite radar for military surveillance and reconnaissance. Amid the uncertainty of localized wars, not only developed countries like the United States, European Union, Japan, and China, but also nations worldwide are increasingly focused on developing satellite communication and radar technology for national security [5,6].

The frequency bands used for satellite communications are primarily divided into C-band, X-band, Ku-band, and Ka-band. Among these, Ku-band and Ka-band offer advantages such as supporting large data transmission, providing high-resolution services with wide bandwidth, and enabling antenna miniaturization due to their shorter wavelengths [4]. However, due to the high operating frequency, the performance of transceiver modules used in towers and earth stations has been limited, making development challenging. Recent advances in complementary metal–oxide–semiconductor (CMOS) and gallium nitride (GaN) high-electron mobility transistor (HEMT) semiconductor process technology have, however, enabled commercialization. Ku-band is now used not only for satellite communication but also in transmitter and receiver modules (TRMs) for unmanned aerial vehicle (UAV) communication, which is increasingly being utilized alongside satellites due to its advantages in miniaturization and bandwidth [7].

**Figure 2:**
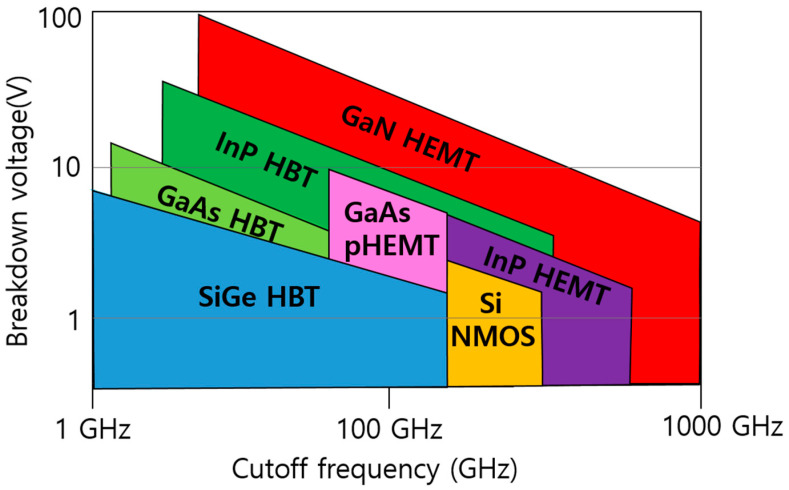
Comparison of breakdown voltage and cutoff frequency among various high-speed semiconductor devices [9,10]. (Data from [9,10]).

Table 1 compares the electrical properties of major semiconductor materials [8], while Figure 2 illustrates the breakdown voltage characteristics as a function of cutoff frequency for semiconductor devices [9,10]. As shown in Table 1 and Figure 2, GaN HEMT semiconductors have been widely studied and commercialized as high-frequency power semiconductors due to their high breakdown voltage and excellent electron mobility stemming from their wide energy band gap. High-power amplifiers (HPAs) present significant challenges in achieving performance in Ku-band satellite and UAV TRMs. To attain high output power, traveling wave tube amplifiers (TWTAs), which utilize vacuum tube technology, have traditionally been employed; however, they are heavy and unstable due to their high-voltage operation. Now, advances in GaN HEMT semiconductor process technology are enabling the replacement of conventional TWTAs with solid-state power amplifiers (SSPAs) [11,12].

In line with this trend, research on Ku-band GaN HEMT HPAs has been actively conducted by various research groups worldwide. This paper aims to summarize the current status and major issues related to Ku-band GaN HEMT HPA technology developed in recent years. The paper is organized as follows: In Section 2.1, we compare the processes used to implement Ku-band GaN HEMT HPAs; in Section 2.2, we discuss the two main approaches to implementing HPAs—monolithic microwave integrated circuits (MMICs) and internally matched power amplifier modules (IM-PAMs)—and examine their respective high-power design strategies and key issues. In Section 2.3, we review research on additional performance enhancements beyond high power, and finally, we provide conclusions.

## 2. Ku-Band GaN HEMT HPA Technology

### 2.1. GaN HEMT Process

**Figure 3:**
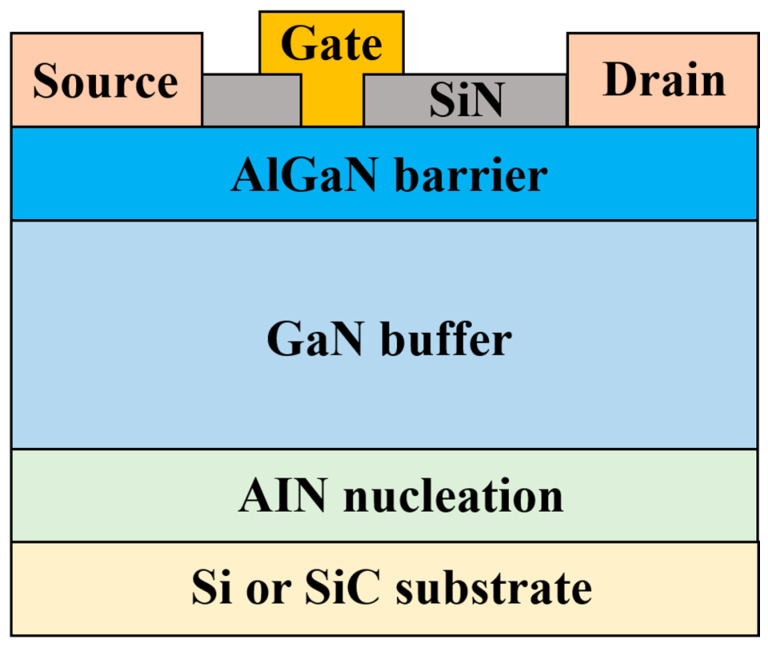
Cross-sectional structure of a typical GaN HEMT [13].

To realize a GaN HEMT power amplifier, it is essential to develop a GaN HEMT process that provides the necessary design kits and fabricates the circuits or devices. Figure 3 shows the cross-sectional structure of a typical GaN HEMT [13]. GaN HEMTs with high cutoff frequency (f_T_) and maximum oscillation frequency (f_MAX_) are required, as obtaining gain becomes more challenging with increasing operating frequency. Therefore, the gate length of the transistor needs to be reduced according to the scaling rule. In this case, the breakdown voltage is also reduced proportionally, which can be a disadvantage in power amplifier design. The substrate material is also an important factor in the choice of process. Generally, GaN HEMT processes are divided into two types: those using silicon carbide (SiC) substrates and those using silicon (Si) substrates. SiC substrates have high thermal conductivity, which is beneficial for dissipating heat generated by self-heating due to the high power density of GaN HEMTs. Therefore, they are often used in the process for GaN HEMT HPAs. However, SiC is hard and difficult to process during post-processing tasks such as dicing. In contrast, Si substrates are easier to produce using mature silicon-based semiconductor technology, are simpler to process, and are more amenable to mass production, providing significant economic advantages. However, they have relatively poor thermal conductivity and are more susceptible to self-heating effects.

The most commonly used processes for GaN HEMT HPAs are the 0.15 μm and 0.25 μm GaN HEMT processes. Here, 0.15 μm and 0.25 μm refer to the gate lengths of GaN HEMT devices. To achieve the required gain for power amplification in Ku-band, GaN HEMTs with gate lengths of 0.25 μm or less are preferred. Recently, 0.15 μm processes have become more popular due to their ability to achieve a high gain and high power added efficiency. Table 2 compares the device and process information for the commercial 0.25-μm GaN HEMT process, while Table 3 compares the device and process information for the commercial 0.15-μm GaN HEMT process.

Most commercial foundries still use SiC substrates, with the exceptions of MACOM/France (formerly OMMIC) and GCS. The 0.25 μm process is based on a drain voltage of 28–30 V. Recently, GaN HEMT processes have been developed to operate at higher voltages of 40–50 V, depending on the foundry, leading to a nearly twofold increase in power density. However, the f_T_ is only 23 to 32 GHz, which can limit power gain at high edge frequencies in the Ku-band. Breakdown voltages range from 75 to 200 V. Meanwhile, the 0.15 μm process utilizes a somewhat lower drain voltage of 20 to 28 V compared with the 0.25 μm process, with a minimum of 12 V for OMMICs using Si substrates. Breakdown voltages for this process range from 50 to 120 V. The lower drain voltage reduces the voltage swing of the power amplifier, which is a disadvantage for achieving high output power. However, the f_T_ performance, which is two to three times higher than that of the 0.25 μm process, is favorable for obtaining high power gain and improving efficiency. As shown in Table 3, the power-added efficiency (PAE) of over 50% at 30 GHz led to the expectation of better power efficiency compared with the 0.25 μm process in the Ku-band. MACOM/France (formerly OMMIC)‘s GaN-on-Si HEMT process has shown output power performance comparable to other processes, even with a drive voltage of 12 V. HPA designs using cascode or stacked-field effect transistor (FET) structures that enhance the voltage swing in this process are expected to yield higher output power and warrant further research [21,22].

### 2.2. High Power Amplifier Design

There are two main ways to implement HPAs. The first is the MMIC type, where both transistors and input/output matching circuits are designed and fabricated within a single integrated circuit [23,24,25,26,27,28,29,30,31,32,33,34,35,36]. This approach reduces the overall circuit size, facilitates mass production, and significantly minimizes parasitic components generated by the connection between the transistor and the matching circuits [20,37]. However, achieving a high dielectric constant ($\varepsilon_{r}$) is challenging with conventional semiconductor processes, making it difficult to use high-Q inductors. Additionally, employing passive elements with large inductance (L) or capacitance (C) values for matching and bias circuits increases costs due to the larger chip size.

The second method is the microwave integrated circuit (MIC) or hybrid type, which utilizes packaged transistors while implementing the input/output matching circuit on the printed circuit board (PCB) [37]. This method leverages PCBs with low losses and high dielectric constants to reduce matching losses by using high-Q inductors and allows for the use of large inductors and capacitors. It also offers the advantage of being tunable after fabrication, enabling easy modifications and optimizations [37]. However, this method results in a bulkier overall circuit size, and matching is limited due to parasitic components caused by wire bonding when connecting the transistor and the matching circuit [37,38]. As frequency increases, the impact of these parasitic components becomes more significant, degrading performance. To address this, the internally matched HPA design is often used, incorporating matching circuitry within the packaged transistors [39,40,41,42,43,44,45,46,47].

Table 4 summarizes the key performance characteristics of reported Ku-band GaN HEMT HPA MMICs. As shown in Table 4, Ku-band power amplifier MMICs have been implemented on silicon and silicon carbide substrates, with output power ranging from 7.2 W to 93 W and gate lengths from 0.1 μm to 0.25 μm. Notably, several papers using the 0.15 μm process have demonstrated output powers exceeding 40 W while achieving power-added efficiencies (PAEs) of 30–40% [24,36]. In references [29,30], high efficiencies close to 40% were also reported at output powers above 10 W. GaN HEMT HPAs implemented with 0.25 μm processes exhibit somewhat lower power efficiency but achieve high output powers of over 40 W, as seen in references [28,32]. A recent paper [31] utilized a 0.2 μm internal process to achieve output power close to 100 W in MMIC form. This demonstrates that MMIC technology has advanced to the point where it can achieve performance comparable to that of existing IM-PAMs, and it is expected to replace TWTAs in the Ku-band.

Table 5 compares HPA MMIC papers with output powers exceeding 20 W from a circuit design perspective. All references adopted a multistage amplifier approach. Except for [32], which was designed as a four-stage amplifier, the other references used a three-stage configuration to drive the main power cell. For output powers up to 20 W, the main power cell combined the currents of eight GaN HEMTs in parallel, resulting in a total gate width of approximately 10 mm. For output powers above 40 W, the standard design approach employed sixteen GaN HEMTs in parallel, with a total gate width ranging from 10 to 25 mm. Notably, Ref. [31] presents a power cell with a total gate width of about 10 mm that achieved an output power of nearly 100 W, demonstrating excellent power density.

**Figure 4:**
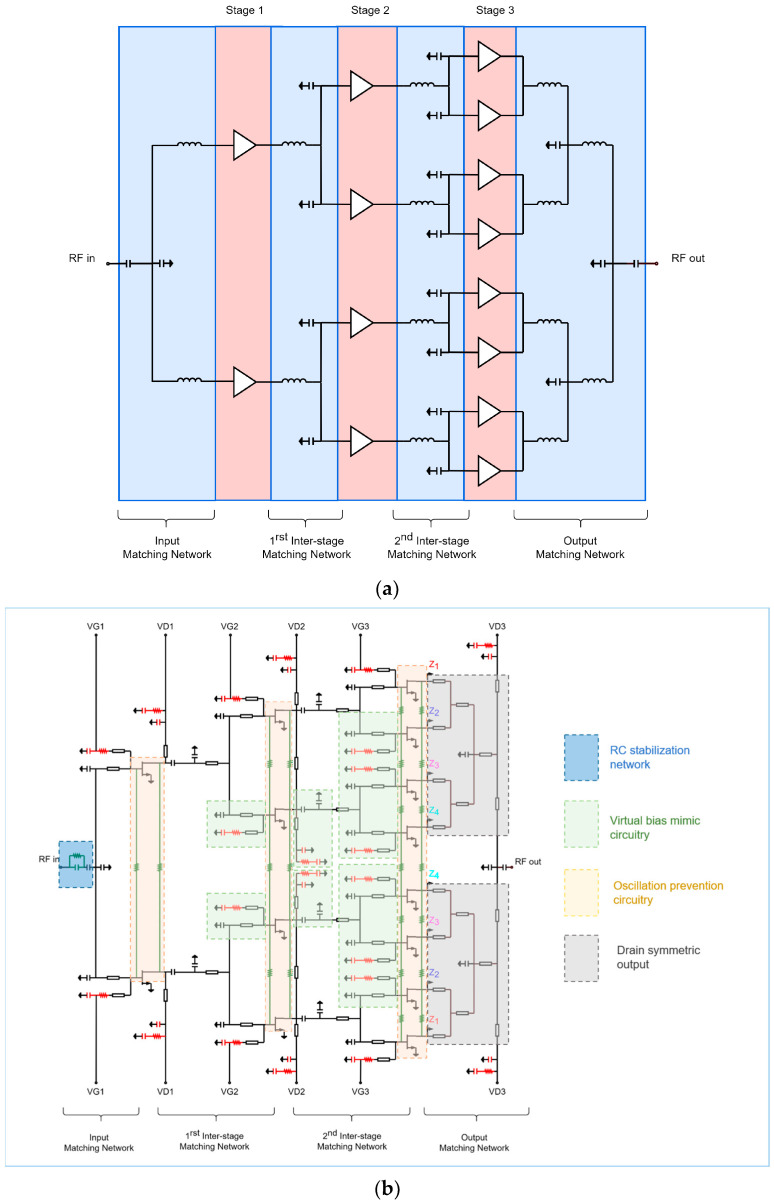
(**a**) Structure and (**b**) circuit schematic of conventional GaN HEMT HPA MMIC. Figures reproduced or reworked with permission from ref. [33]. Copyright 2023 MDPI.

Figure 4a,b show the HPA structure and schematic commonly used in the aforementioned papers. The HPA consists of a two-way GaN HEMT power cell in the first stage, a four-way configuration in the second stage, and an eight-way configuration in the third stage. In contrast, an HPA with a 16-way GaN HEMT as the main power cell replicates the previously described 2-4-8 structure, tying the inputs and outputs together.

As shown in the schematic of Figure 4b, an RC stabilization circuit with resistors and capacitors connected in parallel is often employed to enhance the stability of the input side of the circuit. Due to the large size of the transistor, substantial DC currents and RF signals are input and output. This increases the risk of oscillation, particularly if the signal diverges to one side. To mitigate even-mode and odd-mode oscillations, large resistors are typically connected to the gate and drain (see the beige boxes in Figure 4b) [48].

**Figure 5:**
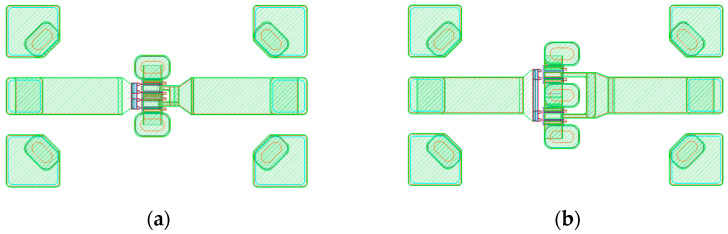
(**a**) OSV and (**b**) ISV layouts of a 4 × 50 μm GaN HEMT.

Another design consideration is the source via the structure of the GaN HEMT. There are two main types of source vias: the outer source via (OSV), where only the outermost part of the source finger is connected, and the individual source via (ISV), which connects ground vias for each of the source fingers. Figure 5a,b illustrate the layouts of a 4 × 50 μm GaN HEMT device implemented in OSV and ISV configurations, respectively. In general, larger and more numerous ground source vias reduce the parasitic inductance caused by the source via structure, which improves load-pull characteristics and enhances device performance by dissipating heat generated by self-heating more effectively. However, the ISV structure can excessively increase the size of the main power cell, posing challenges for MMIC manufacturing. Therefore, it is common to use an ISV structure for the drive stage and an OSV for the main stage [32].

Additionally, careful design is essential for the bias circuit. As shown in Figure 4b, a virtual bias mimic circuit with open stubs and RCs in series on the opposite side of the actual bias input is used to ensure that each connected transistor presents the same impedance to the gate bias supply (see the green boxes in Figure 4b). The drain bias circuit also requires careful design to ensure that each transistor receives a drain voltage through a line that is in phase with the others (see the gray boxes in Figure 4b).

Table 6 compares Ku-band GaN HEMT HPAs implemented using the MIC approach. Compared with the MMIC schemes in Table 4, more 0.25 μm processes were utilized, with the average output power ranging from 50 to 120 W, which was higher than that of the MMIC schemes. However, the PAEs fell within the 20 to 30 percent range. All MIC power amplifiers in Table 6 were implemented in the IM-PAM type.

Table 7 compares the HPA MIC references of Table 6 from a circuit design perspective. The GaN dies used in the surveyed papers were primarily the CGHV1J070D, sold by MACOM (formerly Wolfspeed), and one developed in-house by Mitsubishi, Japan. The number of GaN HEMTs in the main power stage varied from 12 to 64, depending on the output power, with a total gate width approximately in the range of 15 to 30 mm. It is evident that MIC power amplifiers are primarily developed to achieve high output power in a narrow band. Due to the size constraints imposed by PCB implementation of input–output matching circuits, a single-stage design was often utilized, which necessitates a separate high-input driving power in the system configuration.

**Figure 6:**
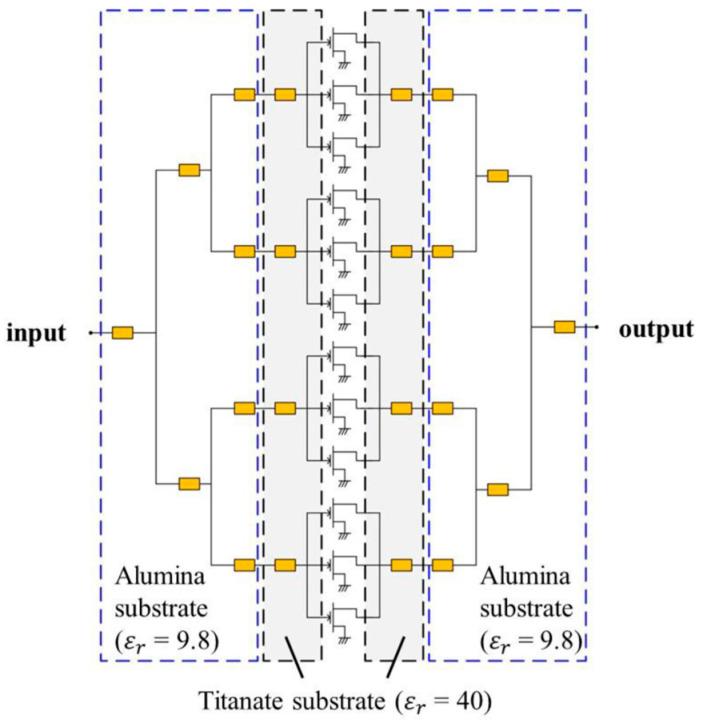
Design example of a Ku-band MIC HPA implemented with an internal matching approach. Figures reproduced with permission from ref. [43]. Copyright 2018 MDPI.

Figure 6 illustrates a typical example of a Ku-band MIC power amplifier implemented using an internal matching approach. To compactly implement an impedance transformer that transformed the low impedance of a large GaN HEMT die into a high impedance, the PCB connected to the GaN HEMT die was made from a material with a very high dielectric constant, as shown in Figure 6. This design prevented the feeding line widths from becoming excessively large, keeping the overall module size manageable. The power dividing and combining components that followed were implemented using materials with lower dielectric constants. In [41], PCBs with $\varepsilon_{r}$ values of 38.5 and 9.8 were used, while [43] also utilized PCBs with $\varepsilon_{r}$ values of 40 and 9.8. When transforming impedance, it is common to employ a multi-step impedance conversion to achieve a gradual transition and avoid a high Q-factor on the Smith chart [43].

**Figure 7:**
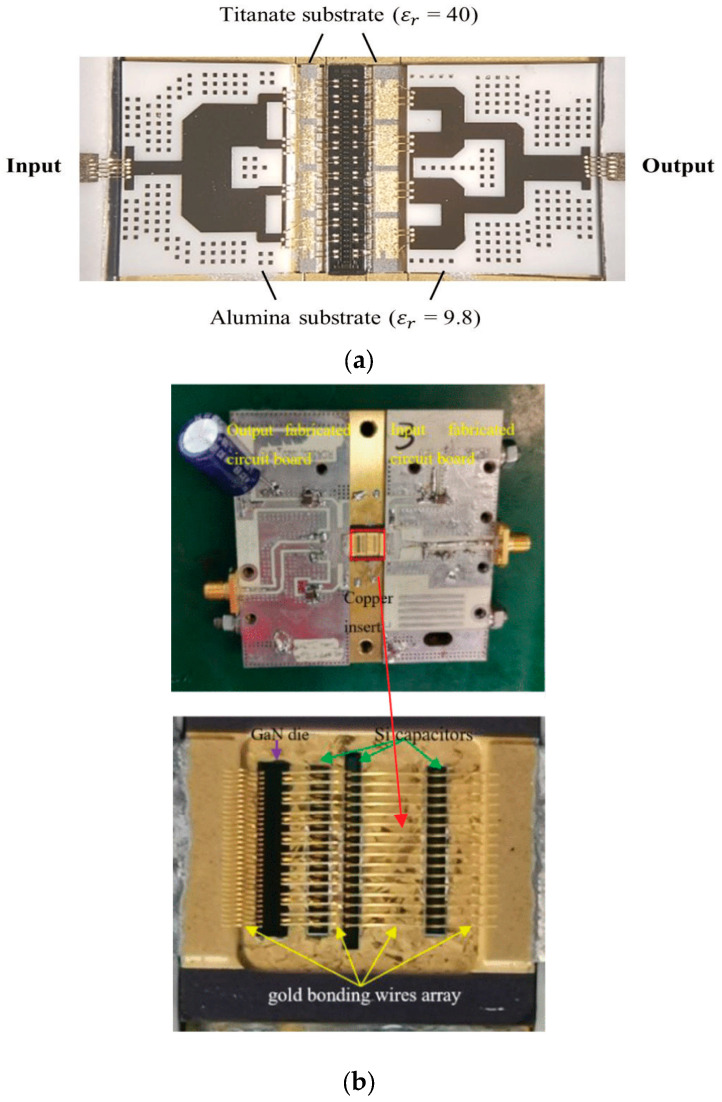
Example photos of (**a**) a fabricated Ku-band GaN HEMT IM-PAM and (**b**) a GaN HEMT power amplifier module with wire bonding. Figures reproduced or reworked with permission from refs. [38,43]. (**a**) is Copyright 2018 MDPI and (**b**) is Copyright 2023 IEEE.

Additionally, the GaN HEMT die and PCB were connected via wire bonding, and this was advantageous to minimize the bonding length and maximize the number of bonds to reduce parasitic inductance. Figure 7a shows an example photograph of an actual fabricated Ku-band GaN HEMT IM-PAM, and Figure 7b presents a photo of a GaN HEMT power amplifier module featuring extensive wire bonding.

**Figure 8:**
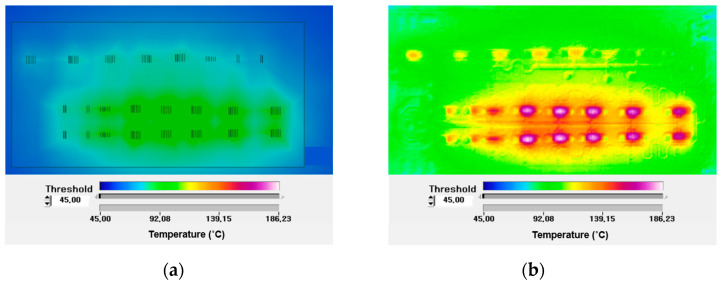
Temperature distribution of a 20 W class GaN HEMT HPA bare die (**a**) with only DC power applied and (**b**) with DC power and RF power applied using a high-resolution IR scope.

On the other hand, packaging and die attach technology that effectively dissipates heat is critical for achieving large output powers of 50 W or more. Figure 8a,b display the temperature distribution of a 20 W class GaN HEMT HPA bare die, with only DC power applied and with both DC and RF power applied, using a high-resolution infrared (IR) scope. It is evident that the maximum temperature nearly doubled from 90 °C to over 180 °C when RF power was applied near the center channel of the transistor, highlighting the necessity for effective heat dissipation techniques during the packaging process.

**Figure 9:**
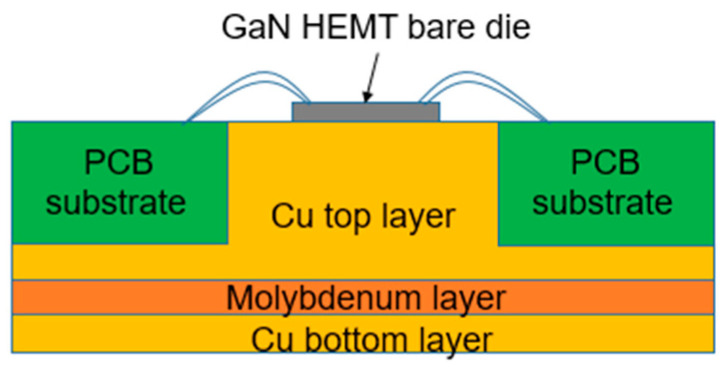
Heat sink structure of a GaN HEMT die [44].

**Figure 10:**
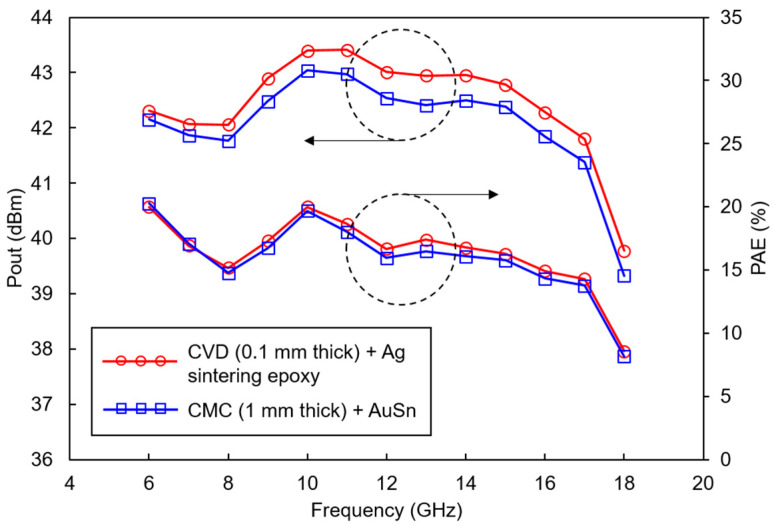
Comparison of output power and PAE of GaN HEMT MMICs according to thermal interface material and heat spreader combinations.

Table 8 compares the thermal conductivity of heat spreader materials commonly used in die attach. Table 9 summarizes the thermal conductivity of materials utilized as heat sinks or thermal interfaces. Several studies have employed eutectic die attachments with good thermal conductivity, mounting them on Cu-Mo-Cu flanges, which serve as excellent heat sinks, rather than the typical copper jig [44]. Figure 9 illustrates the heat sink structure of a GaN HEMT die, frequently used in IM-PAMs. Reference [49] reports improvements in output power and PAE by using chemical vapor deposition (CVD) diamond materials, which possess the best thermal conductivity, as the thermal interface on top of the copper heat sink, as shown in Figure 10.

In this subsection, we investigated two methods for implementing Ku-band GaN HEMT HPAs, MMICs and internally matched MIC design methods, by comparing the reported papers. As mentioned above, MMIC HPAs can be configured in a multistage arrangement with a compact size to achieve high gain, offering a low input power burden and wide bandwidth. In contrast, MIC HPAs are advantageous for obtaining high output power within a narrow bandwidth; however, due to size constraints, they are typically limited to a single-stage configuration and require high input power. Nonetheless, advancements in MMIC processes and design technologies have been significant in recent years, suggesting that we can expect numerous studies reporting output powers exceeding 100 W in the near future, such as in [31].

### 2.3. Other Additional Design Techniques

Ku-band GaN HEMT power amplifiers have been extensively studied to achieve high output power as a replacement for TWTAs. However, several research groups have conducted and published studies aimed at improving performance beyond just output power.

To enhance linearity, some researchers have embedded linearizers within the MMIC or designed separate linearizer modules in front of the MIC [23,47]. In [23], a linearizer composed of a simple diode and the inductance of a microstrip line was placed between the buffer and the power amplifier, resulting in an increase of 5 dB in linear output power with third-order intermodulation distortion (IMD3) levels below −25 dBc. In [47], a linearizer using two diodes, a bandpass filter (BPF), and microstrip lines was designed for the front end of the entire system, including the MIC power amplifier, improving amplitude modulation to amplitude modulation (AMAM) and amplitude modulation to phase modulation (AMPM) by 2 dB and 5 degrees, respectively.

In addition to linearity, a Doherty power amplifier was employed in [50] to increase backoff efficiency for telecommunication systems, achieving drain efficiencies exceeding 28% at 6 dB backoff power. In [25], a control circuit for load modulation was integrated into a balanced power amplifier design, resulting in 10–16 W output power and a high power-added efficiency (PAE) of 25–40% across the 6–18 GHz band, including Ku-band.

With the expansion of Ku-band satellite communication and UAV communication services, further research is anticipated to improve the linearity and efficiency of Ku-band GaN HEMT power amplifiers while maintaining high output power.

## 3. Conclusions

This article reviews the current state of Ku-band GaN HEMT high-power amplifiers for satellite communications, which are actively being developed and researched. Recent MMIC designs focus on GaN HEMT processes with gate lengths less than 0.2 μm on SiC substrates, achieving PAEs exceeding 30% and output powers nearing 100 W. Utilizing advanced heat dissipation packaging from MIC design, Ku-band GaN HEMT amplifiers are anticipated to expand into both GaN-on-SiC and cost-effective GaN-on-Si MMICs. Future developments will focus on enhancing linearity, backoff efficiency, and bandwidth to support applications in military and civilian satellite and UAV communications. Additionally, advancements in power efficiency, thermal management, and miniaturization will enable broader adoption across mobile and satellite platforms, with new markets like 5G/6G expected to drive commercial and defense applications. These trends underscore the ongoing importance of research in Ku-band GaN HEMT HPA technology.

## Figures and Tables

**Table 1:** Comparison of the electrical properties of major semiconductor materials [8].

Property	Si	GaAs	SiC	GaN
Energy Bandgap (eV)	1.11	1.43	3.2	3.4
Critical Electric Field (MV/cm)	0.3	0.5	3.0	3.5
Charge Density (×10^13^/cm^2^)	0.3	0.3	0.4	1
Mobility (cm^2^/V/s)	1350	8000	900	1500
Saturation Velocity (×10^7^ cm/V)	1	1.4	2	2.7

**Table 2:** Comparison of device and process information for commercial 0.25-um GaN HEMT process [14,15,16,17,18,19,20].

Foundry	V_DD_ (V)	Substrate	Breakdown Voltage (V)	f_T_ (GHz)	P_out_ @10 GHz (W/mm)	PAE @10 GHz (%)
Qorvo	40	SiC	75	32	6	>60
MACOM (Wolfspeed)	28/40	SiC	>84	−	4.2/6.6	>55
GCS	28/48	SiC/Si	200	23	4 ^1^/10.8	45 ^1^/65
UMS	30	SiC	>120	25	4.5 ^2^	−
WIN semi	28/40	SiC	120	23	5/10	65/60
NXP	50	SiC	>150	−	−	−

^1^ The data were measured at 15 GHz. ^2^ The frequency was unknown.

**Table 3:** Comparison of device and process information for commercial 0.15-um GaN HEMT process [14,15,16,17,18,19,20].

Foundry	V_DD_ (V)	Substrate	Breakdown Voltage (V)	f_T_ (GHz)	P_out_ @30 GHz (W/mm)	PAE @30 GHz (%)
Qorvo	28	SiC	50	90	4.2	>50
MACOM (Wolfspeed)	28	SiC	84	−	3.75	>40
GCS	28	SiC	100	42	3	55
UMS	20–25	SiC	>80	−	4.2 ^2^	36 ^3^
WIN semi	28	SiC	120	35	5	50
NXP	20–28	SiC	>100	−	−	−
MACOM/France (formerly OMMIC) ^1^	12	Si	>50	150	4 ^2^	48 ^2^

^1^ The foundry uses 0.1 μm GaN HEMT process. ^2^ The frequency was unknown. ^3^ The data were estimated from the graphs.

**Table 4:** Summary of the key performance characteristics of reported Ku-band GaN HEMT HPA MMICs.

Reference	Gate Length (μm)	Substrate	V_DD_ (V)	Pout (W)	PAE (%)
[23]	0.25	−	24	20	16
[24]	0.15	SiC	28	47.5	36.2
[25]	0.25	SiC	25	7.2–9.5	35
[26]	0.25	−	30	25	26–30
[27]	0.15	SiC	25	7.9	35
[28]	0.25	SiC	40	63	30
[29]	0.15	SiC	28	8.3–13.2	35.7–45.4
[30]	0.15	SiC	28	16–25	30–40 ^1^
[31]	0.20	SiC	28	79–93	28.7–31.5
[32]	0.25	SiC	28	40	17
[33]	0.1	Si	11	8.9	27
[34]	0.1	Si	12	8.9–15.8	30–41 ^1^
[35]	0.1	Si	9	10	35
[36]	0.15	−	28	40–50	36

^1^ The data were estimated from the graphs.

**Table 5:** Comparison of reported HPA MMICs above 20 W from a circuit design perspective.

Reference	BW (GHz)	Output Power (W)	# of GaN HEMTs in the Final Stage	Total Gate Width (mm)	# of Stages
[23]	13.75–14.5	20	8	9.6	3
[26]	13–18	25	8	8.64	3
[28]	12.7–13.25	63	16	−	3
[30]	13–17	16–25	8	7.68	3
[31]	14–18	79–93	16	10.88	3
[32]	15.25–16.25	40	16	25.6	4
[36]	13–15.5	40–50	16	15.36	3

**Table 6:** Summary of the key performance characteristics of reported Ku-band GaN HEMT HPA MICs.

Reference	Gate Length (μm)	Substrate	V_DD_ (V)	Pout (W)	PAE (%)
[39]	−	−	24	63	32
[40]	−	−	24	50	29
[41]	0.25	SiC	24	63	25
[42]	0.25	SiC	40	50	23
[43]	0.25	SiC	40	57–66	−
[44]	0.25	SiC	50	50	−
[45]	0.15	−	24	100	32 ^1^
[46]	−	−	24	80	23–28 ^1^
[47]	0.15	−	30	120	31

^1^ The data were estimated from the graphs.

**Table 7:** Comparison of reported HPA MICs from a circuit design perspective.

Reference	BW (GHz)	Pout (W)	# of GaN HEMTs in the Final Stage	Total Gate Width (mm)	# of Stages
[41] ^1^	13.75–14.5	63	24	28.8	1
[42]	13.75–14.5	50	12	14.4	2
[43] ^2^	16.2–16.8	57–66	12	14.4	1
[44] ^2^	12.4–13.8	50	12	14.4	1
[45] ^1^	−	100	48	28.8	1
[46] ^1^	13.75–14.5	80	48	−	1
[47] ^1^	11.7–12.2	120	64	30.72 ^3^	1

^1^ These references used the same GaN HEMT die (CGHV1J070D by MACOM). ^2^ These references used GaN HEMT dies developed in-house by Mitsubishi, Japan. ^3^ The data were estimated from the figures.

**Table 8:** Comparison of thermal conductivity of heat spreader materials. (Data from [49]).

Composition	Thermal Conductivity (W/mK)
PbIn	17
AuGe	44
SnPb	50
AuSn	57
SnAg	78
Ag Sintering Epoxy	100

**Table 9:** Comparison of thermal conductivity of heat sink or thermal interface materials. (Data from [49]).

Composition	Thermal Conductivity (W/mK)
Mo	140
W	170
Al	230
Cu	400
CMC ^1^	270–320
CVD Diamond	1000–1800

^1^ CMC means copper/molybdenum/copper combination composition in equal parts.
